# A multi-centre randomised controlled trial of rehabilitation aimed at improving outdoor mobility for people after stroke: Study protocol for a randomised controlled trial

**DOI:** 10.1186/1745-6215-13-86

**Published:** 2012-06-21

**Authors:** Pip A Logan, Mat P Leighton, Marion F Walker, Sarah Armstrong, John R F Gladman, Tracey H Sach, Shirley Smith, Ossie Newell, Tony Avery, Hywel Williams, James Scott, Kathleen O’Neil, Annie McCluskey, Simon Leach, David Barer, Claire Ritchie, Ailie Turton, Jane Bisiker, David Smithard, Tess Baird, Paul Guyler, Therese Jackson, Ingrid Watmough, Maggie Webster, Janet Ivey

**Affiliations:** 1 Division of Rehabilitation and Aging, University of Nottingham, Nottingham, UK; 2 Nottingham Clinical Trials Unit, University of Nottingham, Nottingham, UK; 3 East Midlands Research Design Service, University of Nottingham, Nottingham, UK; 4 University of East Anglia, Norwich, UK; 5 NHS Nottingham, Nottingham, UK; 6 Stroke Survivor, Nottingham, UK; 7 Division of Primary Care, University of Nottingham, Nottingham, UK; 8 University of Nottingham, Nottingham, UK; 9 Nottingham City Social Services, Nottingham, UK; 10 Gateshead PCT, Gateshead Health NHS Foundation Trust, Nottingham, UK; 11 The University of Sydney, Sydney, Australia; 12 University of Lincoln Hospitals NHS Trust, Lincoln, UK; 13 NHS Lanarkshire, Coatbridge, UK; 14 University of West of England, Bristol, UK; 15 Wolverhampton PCT, Wolverhampton, UK; 16 Kent Community Health Trust, Ashford, UK; 17 Tower Hamlets PCT, London, UK; 18 Southend Hospital NHS Foundation Trust, Southend, UK; 19 NHS Grampian, Aberdeen, UK; 20 Norfolk PCT, Norwich, UK; 21 Cardiff and Vale University Health Board, Cardiff, UK; 22 Cwm Taf Health Board, Mid-Glamorgan, UK

**Keywords:** Stroke rehabilitation, Complex intervention, Randomised controlled trial

## Abstract

**Background:**

Up to 42% of all stroke patients do not get out of the house as much as they would like. This can impede a person’s quality of life. This study is testing the clinical effectiveness and cost effectiveness of a new outdoor mobility rehabilitation intervention by comparing it to usual care.

**Methods/design:**

This is a multi-centre parallel group individually randomised, controlled trial. At least 506 participants will be recruited through 15 primary and secondary care settings and will be eligible if they are over 18 years of age, have had a stroke and wish to get out of the house more often. Participants are being randomly allocated to either the intervention group or the control group. Intervention group participants receive up to 12 rehabilitation outdoor mobility sessions over up to four months. The main component of the intervention is repeated practice of outdoor mobility with a therapist. Control group participants are receiving the usual intervention for outdoor mobility limitations: verbal advice and provision of leaflets provided over one session.

Outcome measures are being collected using postal questionnaires, travel calendars and by independent assessors. The primary outcome measure is the Social Function domain of the SF36v2 quality of life assessment six months after recruitment. The secondary outcome measures include: functional ability, mobility, the number of journeys (monthly travel diaries), satisfaction with outdoor mobility, mood, health-related quality of life, resource use of health and social care. Carer mood information is also being collected.

The mean Social Function score of the SF-36v2 will be compared between treatment arms using a multiple membership form of mixed effects multiple regression analysis adjusting for centre (as a fixed effect), age and baseline Social Function score as covariates and therapist as a multiple membership random effect. Regression coefficients and 95% confidence intervals will be presented.

**Discussion:**

This study protocol describes a pragmatic randomised controlled trial that will hopefully provide robust evidence of the benefit of outdoor mobility interventions after stroke for clinicians working in the community. The results will be available towards the end of 2012.

**Trial registration:**

ISRCTN58683841

## Background

Stroke can have a devastating effect on people's lives, with half of survivors being dependent on others six months later [1], one third feeling socially isolated, one quarter having abnormal mood and half not getting out of their house as much as they would like. These are worldwide health care issues which affect many people. A total of 130,000 people a year have a stroke in England and Wales (Office of National Statistics 2001). Community based research over the last three decades has shown that people living at home with stroke have felt neglected, not provided with the correct information and have received patchy levels of rehabilitation [2]. The NHS has developed guidelines through the National Service Framework for Older People [3] and the National Clinical Stroke Guidelines [4] to help commissioners and therapists provide evidence-based practice. However, within these documents there is very little information about the long-term rehabilitation that should be provided because the research evidence is not available to help make robust recommendations.

A single centre randomised controlled trial which evaluated an outdoor mobility rehabilitation intervention found clear outdoor mobility benefits for those people who received the intervention [5]. The generalisability of the study was limited as the intervention was delivered by a single therapist, in one city and there was no economic evaluation. Our single centre trial provided confirmation that we were asking a relevant and important question and that the intervention could be implemented within existing NHS structures. This present multi-centre trial was planned to evaluate the clinical and economic effectiveness of the intervention before we can recommend its wider adoption.

## Methods/design

### Trial purpose

The trial’s purpose was to confirm or refute the clinical effectiveness and cost effectiveness of treating stroke patients with outdoor mobility limitations with a novel targeted rehabilitation therapy intervention versus standard clinical practice.

### Trial objectives

The health care objective is to improve the quality of people’s lives after a stroke by enabling them to get out of the house more often and when they wish.

The research objective is to test the effectiveness and cost effectiveness of treating people who have had a stroke with a new outdoor mobility rehabilitation intervention.

### Trial design

This is a multi-centre parallel group randomised, controlled trial. The trial consists of an initial baseline visit to provide information and explain the study, take informed consent, provide the routine clinical stroke rehabilitation treatment, complete a set of questionnaires and provide a travel diary with instructions. Randomization to the control or intervention group is stratified by age (<65 years and ≥65 years) and site. The intervention group are receiving up to 12 intervention visits within four months from recruitment. All participants are completing monthly travel diaries and questionnaires at 6 and 12 months.

### Site and participant recruitment

We planned to have 15 NHS community or Primary or Secondary Care Trust study sites throughout England, Scotland and Wales. People who have had a stroke were identified between November 2009 and July 2011 through GP practices, primary care therapy teams and community stroke teams. A letter along with study information was sent to the identified stroke patients by a member of their normal clinical care team, asking if they were interested in taking part in the study. The letter contained a reply slip and a reply paid envelope. If the potential participant was interested in taking part, they completed the slip and returned it in the envelope directly to the local stroke/therapy team, who then informed the local Research Assistant (RA). The RA then contacted the patient to arrange an appointment to discuss the study further and respond to any questions. If needed, an interpreter, translator or signing service was available. The RA explained the details of the trial and provided a Participant Information Sheet, ensuring that the participant had sufficient time to consider whether or not to participate.

### Informed consent

It was explained to the potential participant that their entry into the trial was entirely voluntary and that their treatment and care would not be affected by their decision. It was also explained that they could withdraw at any time. In the event of their withdrawal, it was explained that their data collected so far could not be erased and would be used in the final analyses. If they agreed to participate in the study, the RA asked the participant to give written consent before they underwent any interventions related to the study that would not have formed part of their normal care. Those participants unable to sign the consent form were asked to mark the form and a witness (either Carer or other person present) also signed the relevant section on the consent form. One copy was kept by the participant, one by the Investigator, and a third retained in the patient’s GP records. With the consent of the participant the research team informed the participant's GP about their participation in the clinical trial.

### Inclusion criteria

Patients were eligible for the study if they provide written informed consent and if they: (i) are aged 18 years or over; (ii) have had a stroke at least 6 weeks previously and (iii) wish to get out of the house more often.

### Exclusion criteria

Patients were not eligible for the study if they: (i) were not able to comply with the requirements of the protocol and therapy program, in the opinion of the assessor; (ii) still in post-stroke intermediate care or active rehabilitation or (iii) were previously enrolled in this study.

### Outcome measures

#### Baseline

The RA collected basic demographics required for randomisation from each participant at the baseline visit. The baseline assessment questionnaires were completed while the RA was present and returned to the trial coordinating centre. The basic demographics, including the NHS number were recorded and supplied to the NHS IC/NHS Central Register to allow mortality checks prior to follow-up.

#### Assessment questionnaires

The 6- and 12-month assessment questionnaires are being posted to the participants along with reply paid envelopes. If the assessments have not been returned within 2 to 3 weeks of the 6- or 12-month timeline then the participant is contacted by telephone by a member of the research team to remind them. Finally, if two to three weeks later the assessments have still not been returned, then the blinded-to-intervention RA arranges to visit the participant and assist in completing the questionnaires. Using this method, a 90% return rate of active participants (that is, those which had not withdrawn, died or been lost to follow-up) was found in the previous single centre trial with the further 10% collected by an independent assessor.

#### Travel diaries

A travel diary is being completed by all participants on a monthly basis. This is in the form of a calendar with the participants entering the number of journeys made on the calendar for that particular day. In addition, in order for the study team to assess safety, participants indicate when they have had a fall on a particular day. The participant is provided with 12 calendar months’ worth of travel diary. Training is provided at baseline assessment in the completion and return of the travel diaries. At the end of each month participants are asked to return that month of the calendar to the research team. A reminder letter is sent to the participant, along with a reply paid envelope, a short time before the previous month’s diary is due for return.

#### Primary outcome measure

The social function domain score of the SF-36v2 [6] measured at six months. This is one of the eight domains of the SF-36v2 and is scored and transformed on to a scale 0 (worst possible health state) to 100 (best possible health state).

The secondary outcome measures at 6 and 12 months are: (i) Nottingham Extended Activities of Daily Living Scale [7] (a measure of functional ability). (ii) Rivermead Mobility Index (a measure of mobility) [8]. (iii) Travel Diary (number of journeys). (iv) Yes or no question: "Do you get out of the house as much as you would like?" (Satisfaction with outdoor mobility). (v) General Health Questionnaire 12 (GHQ-12) [9] (measure of participant mood). (vi) GHQ-12 [9] (measure of Carer mood).(vii) EQ-5D [10] and SF-6D (subset of SF-36 v2 [6]) (viii) Resource use of health and social care and provision of equipment. (ix) Participant mortality (death data) will be collected from NHS IC/NHS Central Register.

#### Carer assessment

Part of the study is to assess if any benefits to the participants are allied with benefits to their carer. For this study, the definition of a carer is “someone who provides unpaid care by looking after a family member, friend or partner following stroke”. In order to assess this, we are asking the carer, if applicable, to complete the GHQ-12 questionnaire at baseline, 6 months and 12 months. The RA first asks the participant if they would like their carer to be asked about completing this questionnaire. We provide a carer information sheet, though we do not ask them to provide informed consent as completion of the questionnaire will be seen as implied consent. This is explained to the carer.

### Health economics

The consequences in terms of patient-specific resource use will be measured using responses to the 6- and 12-month assessments; this will capture information on quantity of primary and secondary health care use, use of personal social services and some patient/carer costs.

Unit costs will be derived from routine published cost data sources, such as the PSSRU health and social care costs [11] and NHS Reference Costs [12] using a common price year. This will enable the overall management cost of treatment to be calculated for each patient and, in turn, the incremental mean cost associated with the intervention compared to control to be estimated. A cost utility analysis will be undertaken measuring health-related quality of life using both the EuroQol EQ-5D [10] and SF-6D [6].

### Randomisation

Randomisation is based on a computer generated pseudo-random code using random permuted balanced blocks of randomly varying size, created by the Nottingham Clinical Trials Unit (NCTU) in accordance with their standard operating procedure (SOP) and held on a secure server. The randomisation is stratified by age (<65 years and ≥65 years) and site. Sixty-five years of age was chosen because Mobility Allowance, a monetary allowance is only available under this age and is often used to purchase a car. This is not available for people over 65 years of age. All participants are given the control group standard information prior to randomization in order to minimize performance bias and reduce contamination. Participants are randomly allocated (1:1 ratio) to either the intervention or to the control group. Access to the sequence is confined to the NCTU Data Manager (who is independent from the study team). Investigators/sites access the treatment allocation for each participant by means of a remote, secure, internet-based randomisation system developed and maintained by the NCTU. The sequence of treatment allocations is concealed until interventions have all been assigned and recruitment, data collection and all other trial-related assessments are complete. After confirming study eligibility and obtaining informed consent, the investigator enters basic demographic details into the web-based randomization program. Once these details have been entered irrevocably into the program, the group to which the patient is randomly allocated is provided to the investigator.

### Minimization of bias and maximising blinding

We have taken every step to maintain blinding for the outcome assessments by using postal questionnaires and RAs. Following any visits, at 6 and 12 months, to assist with questionnaire completion, the RA completes a blinding questionnaire to assess if the blinding has been broken. The data are entered, stored and managed by the NCTU until the end of the trial. We are recruiting participants from numerous sites across the UK to reduce response bias and we are using numerous rehabilitation staff to reduce expertise bias.

### Maintenance of randomisation codes and procedures for breaking code

Neither the participants nor the therapist are blinded to which treatment the participants receive. The RA is blind, though there is no foreseeable situation whereby they would need to know the treatment allocation of a particular participant. The outcome assessors are blind, but again there is no requirement for them to know the treatment allocation at any stage. As a result there is no procedure in place for breaking the randomisation code. The statistician analysing the data is also blind and allocations will not be revealed until all data analyses have been completed.

### Duration of the trial

The recruitment phase of the trial is estimated to last a total of three years, from initiation of the first site to completion for the last patient. This commenced in November 2009. For any given participant the duration of their involvement will be a minimum of 12 months to a maximum of 14 months. Participants’ involvement in the trial will end when they have completed and returned the 12-month assessment questionnaires and travel diary.

### End of the trial

The end of the trial is defined as completion and return of the 12-month assessment questionnaires, scheduled to occur within 2 months after the 12-month assessment date of the last participants.

### Removal of participants from therapy or assessments

No specific withdrawal criteria have been defined for this study. If a participant leaves the study prematurely (that is, prior to completion of the protocol), the primary reason for discontinuation will be determined and recorded, if at all possible. Withdrawn participants are not being replaced. Anonymity of participants will be assured.

### The interventions

A novel rehabilitation technique (intervention) group will be compared to a usual care (control) group.

#### Intervention group

Participants receive what is considered clinically to be routine intervention for outdoor mobility limitations. That is, verbal advice and provision of leaflets provided during the baseline assessment visit. In addition, participants in this group receive up to 12 rehabilitation outdoor mobility sessions of about an hour each over four months. Development of this intervention followed the guidelines for complex interventions [13] using qualitative research findings [14] in the pre-clinical stage to develop ‘barriers and need’ theories. The intervention aims to increase outdoor mobility participation by alleviating physical difficulties, develop skills to maximize the individual's potential and to overcome psychological barriers. A description of the intervention has been published [15]. The main component of the intervention is that therapists go repeatedly with patients to try outdoor mobility, including buses, taxis, walking, voluntary drivers and mobility scooters until they feel confident to go alone or with a companion. The number of interventions depends entirely on the participant. If they feel they do not require any further interventions, for whatever reason, then the interventions can stop. If they feel they require additional intervention, for whatever reason, they can continue the intervention, up to a maximum of 12 visits.

#### Control group

Participants receive what is considered clinically to be routine intervention for outdoor mobility limitations. That is, verbal advice and provision of leaflets provided during the baseline assessment visit.

### Sample size and justification

We intend to recruit 506 participants, 253 into each arm.

Our sample size calculations are based on the primary outcome measure, the Social Function domain of the Short Form 36 version 2 (SF-36v2) at six-month follow-up. A recent study suggested a minimally, clinically important difference (MCID) for the social function domain is 12.5 points [16]. Assuming a power of 90% and a two-sided significance level of 5%, we estimate that to detect a difference in mean SF-36v2 scores of 12.5 points assuming a common standard deviation of 28.2 [17], a sample size of 135 patients per arm is required. This calculation assumes an attrition rate of 20% over the six-month period. Clustering by delivery of treatment was allowed for, using an ICC of 0.02 (obtained from a personal communication from Walters) and a centre effect ICC of 0.04 (obtained from Aberdeen University’s database of ICCs (http://www.abdn.ac.uk/hsru/epp/iccs-web.xls)).

Originally, we calculated our total sample size, which was 676 participants, using the same procedure as above but with seven sites. Due to recruitment being slower than anticipated, we extended the recruitment period and increased the number of centres to 15. Revised sample size calculations were based on a number of scenarios. These calculations indicated that a sample size of 440 would have a power of 86% (assuming no attrition) and a power of 82% (assuming an attrition rate of 20%) to detect a difference in means of 12.5 on the Social Function domain of the Short Form 36 version 2 (SF-36v2) at six-month follow-up if there were 7 centres and, if there were 12 or more centres, the corresponding power would be at least 90% if an attrition rate of 20% was assumed. By increasing the number of centres to 15 we were able to use the later calculations.

Our sample size calculations were based on a number of assumptions and although we used the best estimates we could obtain there was some uncertainty about the true values of the ICCs used to adjust for clustering. We, therefore, decided to be cautious and aimed to recruit a sample size of 506 (440 +15%) to ensure we recruited enough participants.

### Statistical analysis

Data are stored in a Microsoft Access Database and analyzed using the statistical package STATA (StataCorp, Texas, USA). Paper records are kept in a locked cabinet, separate from any identifiable information, such as the consent forms. A full data analysis plan has been produced and agreed with the Trial Steering Group. Outcome measures are recorded at 6 and 12 months but the primary time point of interest is 6 months. However, this data will not be analysed until all study data have been collected. The study report will be produced according to the CONSORT recommendations [18].

#### Descriptive analyses

Continuous data that are approximately, normally distributed will be summarised in terms of the mean, standard deviation and the number of observations. Skewed data will be presented in terms of the median, lower and upper quartiles, minimum, maximum and number of observations. Categorical data will be summarised in terms of frequency counts and percentages.

#### Comparisons between treatment arms

Analyses will be undertaken on an intention to treat basis in that participants will be analysed in the group to which they were randomised regardless of whether they received the intervention or not. Two-sided tests will be used to test statistical significance at the 5% level.

### Assessment of efficacy

#### Analysing primary outcomes

The mean Social Function score of the SF-36v2 will be compared between treatment arms using a multiple membership form of mixed effects, multiple regression analysis adjusting for centre (as a fixed effect), age and baseline Social Function score as covariates and therapist as a multiple membership random effect. Regression coefficients and 95% confidence intervals will be presented. The robustness of these findings will be assessed by repeating the analysis and including baseline variables (such as gender) that are associated with the outcome variable as covariates in the model.

#### Analysing secondary outcomes

The outcome variables mobility measured with the Rivermead Mobility Index [8] number of outdoor journeys taken, functional ability measured by the Nottingham Extended Activities of Daily Living Scale [19]; mood and carer psychological distress measured by the General Health Questionnaire 12 [9] will be analysed using the same method as for the primary outcome measure. In all analyses, an adjustment for centre, therapist effect, age and baseline value will be made.

The proportion of participants saying they get out of the house as often as they would like will be compared between treatment arms using a mixed effects logistic regression analysis adjusting for centre (as a fixed effect), age and whether or not they get out of the house as often as they would like as recorded at baseline. Odds ratios and 95% confidence intervals will be presented.

The robustness of the findings for all secondary outcomes will be assessed by repeating the analysis and including baseline variables, (such as gender), that are associated with the outcome variable as covariates in the model.

We acknowledge the potential for type 1 errors associated with significance testing for multiple end points. We will, therefore, consider our analyses of the secondary outcome measures to be partly exploratory in nature, and partly confirmatory of our findings for the primary outcome measure.

### Health economic evaluation

An economic evaluation will be a major element of the project. This component of the study aims to extend the evidence base by estimating for the first time the incremental cost-effectiveness of an outdoor mobility rehabilitation intervention, compared to usual care, from a health and personal social services perspective for the trial period. In addition, the patient and carer perspective will be examined separately.

The EQ-5D includes five pertinent dimensions, (ability to perform usual activities, mobility, anxiety and depression, pain and self-care), each with three levels of severity (no problems, some/moderate problems and severe/extreme problems). Responses to these five dimensions will be converted into one of 243 different EQ-5D health state descriptions, which range between no problems on all five dimensions (11111) and severe/extreme problems on all five dimensions (33333). We will use the York A1 tariff to assign a utility score to each of these states. The SF-6D is measured using the responses to 11 of the questions on the SF-36 questionnaire for use in cost utility analyses [20]. The SF-6D is composed of six dimensions (physical functioning, role limitations, social functioning, pain, mental health and vitality), which have between four and six severity levels. It, thereby, has 18,000 potential health states and scores for these states have been estimated [6]. Both measures are being used at baseline, 6 and 12 months. Quality Adjusted Life Year (QALYs) will be estimated over the trial period between the comparator interventions in order to provide a cost per QALY for the getting out of your house intervention. As a generic measure of outcome this analysis will enable the results to be compared to that of other economic evaluations for stroke and other conditions, in order to assess whether allocation of resources to a getting out of the house intervention offers value for money compared to other potential uses of the resources.

As the EQ-5D and SF-6D utility measures are based on both different health state descriptions, and use different valuation techniques, they can produce different utility scores for the same group of patients. This study will seek to investigate which of these two outcome measures is appropriate using certain published criteria and to explore the impact choice of utility measure has on estimates of cost-utility since further research has been argued as justified in these areas.

Although not anticipated, censored data will be adjusted using appropriate published techniques [21] should this be necessary. If non-dominance occurs (that is, if costs are greater and the intervention is more effective or if the intervention is cheaper and less effective) an incremental cost-effectiveness ratio (the ratio of change in cost divided by change in benefit) will be produced. The confidence region around the incremental cost effectiveness ratio will be estimated using appropriate statistical techniques, such as the non-parametric bootstrap method. This stochastic analysis will enable a cost effectiveness acceptability curve to be produced illustrating the uncertainty surrounding the optimal decision. Probabilistic sensitivity analysis will be undertaken to test the robustness of the results. Estimates of the incremental cost, incremental costs effectiveness and the uncertainty around these estimates will enable us to examine whether "The getting out of the house intervention" offers value for money.

### Fidelity of treatment

The rehabilitation staff providing the treatment are completing an intervention record for each participant and recording number and duration of treatment. These records are monitored against a predefined checklist to make sure the intervention is being delivered according to the protocol. In addition, a selection of therapy visits are observed and scored against a predefined checklist.

### Assessment of safety and adverse events

The main risk associated with this intervention is an increased risk of falling, as participants are being encouraged to become more mobile. So, as all falls will be self-reported on the travel diary, the only adverse events we will record will be any falls within the intervention group that required the assistance of a healthcare professional. To capture these events the therapist will ask, subsequent to the first visit, “Have you had any falls since my last visit?” The occurrence of a serious adverse event as a result of participation within this study is not expected so no serious adverse event data will be collected.

### Participant removal from the study due to adverse events

Any participant who experiences an adverse event may be withdrawn from the study at the discretion of the Investigator.

### Procedures for missing, unused and spurious data

Initially a complete case analysis will be performed for the primary and secondary outcome measures and a sensitivity analysis will then be performed in which missing dependent and independent variable data will be replaced using multiple imputations. Missing data will be imputed separately for the intervention and control groups.

### Compliance

Compliance in this trial will be completion and returning of all diaries and questionnaires. In practice this means 12 monthly travel diaries and three sets of assessment questionnaires, one each at baseline, 6 months and 12 months.

### Criteria for terminating trial

The study may be stopped as a whole because of a change in opinion of the REC or overwhelming evidence of efficacy/inefficacy, safety concerns or issues with trial conduct at the discretion of the Sponsor. Recruitment at a centre may be stopped particularly for reasons of low recruitment, protocol violation or inadequate data recording.

### Trial management

The trial will be managed by the Trial Management Group. The Trial Steering Committee and Data Monitoring Committee will be combined as a risk assessment has been completed and as the risk to participants is low we consider that the two committees will work best if they are merged. This will be called the Trial Steering and Data Committee (TSDC). The TSDC will meet independently prior to the start of the study and will agree to terms of reference and will monitor unblinded data and the conduct of the study. Only the TSDC will have access to unblinded data until the final outcome assessment has been completed. The TSDC will recommend discontinuation of the study if significant ethical or safety concerns arise or if there is very clear evidence of benefit (clinical or statistical) prior to completion of the study. The sponsor is the University of Nottingham, which will clarify with the funding body (the HTA) and local centre research and development departments their precise responsibilities. The CI, delegated by the Sponsor, is responsible for the proper conduct and management of the trial.

### Definition of a protocol deviation

A protocol deviation is an unanticipated or unintentional divergence or departure from the expected conduct of a study inconsistent with the protocol, consent document or other study procedures. Violations of eligibility criteria and other deviations from protocol will be assessed by TMG and discussed with the TSC during study evaluation meeting before data lock and unblinding.

This summary paper is based on version 6.0, 11 February 2011. A copy of the full protocol is available on request.

## Trial status

Participant follow-up is on-going. Final follow-up is the end of August 2012.

## Abbreviations

CI: Chief Investigator; DMC: Data Monitoring Committee; GHQ: General Health Questionnaire; GP: General Practice; HTA: Health Tecnology Assessment; ICC: Intraclass correlation coefficient; MCID: Minimally clinically important difference; NCTU: Nottingham Clinical Trials Unit; NHS: National Health Service; NHS-IC: National Health Service Information Centre; NIHR: National Institute of Health Research; QALYs: Quality adjusted life years; RA: Research Assistant; TMG: Trial Management Group; TSC: Trial Steering Group; TSDC: Trial Steering and Data Committee.

## Competing interests

None of the authors have any competing interests.

## Authors’ contributions

PL conceived the study, is the grant holder and chief investigator for the study, and wrote this summary and the original protocol. ML, based in the Nottingham Clinical Trials unit (NCTU), is responsible for day to day running of the trial and data collection/management and has been responsible for most revisions of the protocol incorporating suggestions and comments from PL, MW, JG, AA, HW, ON, SA, TS, JS, SS, KO and AM. MW, JG, AA, HW, ON, SA, TS, JS, SS, KO, AM, Sl, DB, CR, AT, JB, DS, TB, PG, TJ, IW, MW and JI have contributed to the writing of this summary and helped write the original full protocol. HW, as director of the NCTU, with MW, JG, TA helped PL design the study and secure funding. In addition, SA is the study statistician and has particular responsibility for the analysis. Also, TS is the study health economist and will be responsible for economic analysis. All authors have read and approved the final manuscript.
